# Life for the Premature Baby

**Published:** 1952-10

**Authors:** Beryl Corner

**Affiliations:** Paediatrician, United Bristol Hospitals


### LIFE FOR THE PREMATURE BABY

BY

BERYL CORNER, M.D.
*Paediatrician, United Bristol Hospitals*

Hippocrates in 460 B.C. stated "that no foetus coming into the world before the seventh month of pregnancy can be saved", but history relates the premature birth of a number of famous people including Isaac Newton, Napoleon, Darwin and more recently Winston Churchill. There is however practically no reference to prematurity or its causes in obstetric or paediatric literature prior to this century, except in Paris where the first modern infant incubator was installed by Tarnier in 1880 at the Maternity Hospital. It was big enough to hold several children and designed from a chicken incubator by the Director of the Zoo.

Prematurity is now one of the outstanding paediatric problems and it is an important reflection on advances in Child Health that this is essentially a problem of the last two decades. The lack of previous interest in the subject is little wonder when it is realized that 250 years ago, when the City of London Lying-in Hospital was opened, 1 in 15 of all infants born alive died before leaving the hospital, and only fifty years ago in 1899, 163 of every 1000 infants born alive in this country died before reaching the age of one year. In those days the death of the feeble and immature was merely part of the process of survival of the fittest.

Our knowledge of the incidence of prematurity has been very indefinite, because, until 1944, statistics have been available in this country only from hospitals and death certificates. In 1944 it became compulsory to add the weight of the baby to the birth notification, a measure designed entirely to give information concerning prematurity. Definition of prematurity by calculation of gestation period is subject to so many fallacies that it has been difficult to compare results of infant care; therefore in 1937, the International Committee at Geneva decided to adopt a weight standard, which had already been suggested in the United States. Since that date, a baby is classed as Immature or Premature if it weighs $5\frac{1}{2}$ lb. or less at birth, irrespective of the period of gestation.

Interest in the Premature Infant was really awakened in America, when, under the influence and inspiration of Bundersen, Julius Hess opened the first Premature-Infant station at the Michael Reese Hospital in Chicago in 1922, to study premature infants and to train women in their care. In this country, in 1930, Mary Crosse was put in charge of a small ward for premature infants at the Sorrento Maternity Hospital, Birmingham. Largely as a result of the reports of these workers, in 1944, the Ministry of Health authorized local authorities to make special provision for the care of premature infants.

Statistics will show why this is the most serious problem in Child Health to-day.

TABLE I

NEONATAL MORTALITY

*Neonatal Mortality Rates for Premature and Full-Term Infants*

| Place | Number of live births | Per cent Prem. | Neonatal Mortality | | |
|---|---|---|---|---|---|
| | | | Premature (per 1000 prem. live births). | Full-term (per 1000 full-term live births) | Total |
| Edinburgh Maternity Hospital 1939–40 | 4,886 | 9·1 | 314·0 | 21·8 | 48·3 |
| Aberdeen Maternity Hospital 1941–42 | 3,156 | 11·1 | 391·0 | 18·9 | 60·2 |
| Birmingham City 1947–50 .. .. | 82,360 | 7·2 | 156·0 | 8·4 | 19·5 |
| Bristol City 1947–50 .. .. | 31,675 | 6·5 | 155·0* | 7·9* | 16·5* |

* 1949 & 1950

(Birmingham figures have kindly been supplied by Dr. V. M. Crosse)

In addition to this neonatal mortality, evidence is accumulating that the mortality and morbidity rates in the next eleven months of life may be higher in the premature infant unless special care is provided. Altogether it has been estimated that 15,000 lives may be lost annually in England and Wales from prematurity.

Theodore Harris in 1742 in his treatise on diseases in infancy wrote: " If therefore we are desirous of laying any firm foundation for the care of infants, we must in the first place have our eyes on their natural tenderness and weakness." In 1947 De Lee in his textbook of obstetrics wrote: " The management of premature babies is far different from the care given full-term babies."

What are the " natural tendernesses and weaknesses " of the premature infant? Firstly there is the problem of respiration; asphyxia and atelectasis are the most frequent causes of death. The factors involved are immaturity of the respiratory centre, causing it to be easily depressed by analgesia and poorly responsive to normal stimulation; feeble musculature of the chest wall; immaturity of lung tissue, particularly lack of elastic tissue; small air tubes which easily become obstructed by inhaled liquids; deficiency of the enzyme, carbonic anhydrase, which is necessary for liberation of carbon dioxide and uptake of oxygen from the lungs. Closely associated with asphyxia is the high incidence of intracranial damage due to too easy moulding of the delicate immature tissues and an increased tendency to bleed. In addition labour is often precipitate.

The maintenance of adequate body temperature is a very big problem. Heat loss is excessive owing to large surface area, but heat production is low and the central mechanism for heat regulation functions poorly. Hence the infant becomes cold, lethargic, often refuses to feed and dies if untreated. Oedema is usually associated with asphyxia but is much aggravated when the baby is allowed to get cold. Immaturity of the liver causes marked physiological jaundice in a high proportion of infants and the immature kidneys may lead to problems in maintenance of water and electrolyte balance.

Infection for the premature infant is never trivial, resistance is so low that a fatal bronchopneumonia or meningitis may develop unsuspectedly and cause death rapidly.

Once the problem of asphyxia has been overcome, skilful handling of the feeding is required. Because of the need for rapid growth, proportionately more food is required than in the full-term infant. The capacity to suck and swallow is poor and regurgitation and vomiting occur with great ease, so that inhalation of food is a constant danger, and filling of the stomach too rapidly tends to embarrass respiration. Diarrhoea and abdominal distension are easily induced by unsuitable feeding mixtures. Since storage of vitamins and iron is poor and growth needs are great, vitamin deficiency and anaemia are common unless prevented.

What therefore is involved in the special care of these infants?

The first essential is the closest team-work between the obstetrician and midwife responsible for the labour, the paediatrician and the premature-infant nurse. Skilful use of minimal analgesia and large episiotomy for delivery help to prevent asphyxia. A hot room and heated cot to 94° should be ready for the infant. The air passages are rapidly cleared before the child takes its first breath, and oxygen, which is the greatest stimulant to respiration in the newborn, must always be available. A pale, limp baby may become pink and vigorous very rapidly after being placed in an oxygen tent. During the first twelve hours careful watch is kept on the body temperature and respirations, but otherwise the child should be left in its hot cot without being bathed, dressed or fed. For the small infants special oxygen incubators with temperature up to 100° may be used instead of the routine draught-proof heated cot and oxygen hood; a moistened atmosphere is necessary and the room temperature should be kept constant. Strict barrier nursing technique with individual equipment for the baby helps to prevent infection, and skin cleaning with sterile oil is reduced to a minimum. No tight wrappings or heavy blankets must be employed because they restrict body movement, particularly that of the chest wall. Provided that they are kept warm, the babies benefit greatly from being nursed completely naked. Feeding is not usually begun until the infant shows a desire—rarely before 12 hours and often not for 24–36 hours; all small infants do best with tube feeds, and lethargic bigger babies may require some tube feeding. The first fluid should always be boiled water until the infant's reactions to feeding are evident. Slowly-increasing quantities of human milk are fed to the child until by the fourteenth day, an average of 3 oz. fluid per lb. body weight is given daily. This high feeding should be continued indefinitely according to the child's appetite. Since no food superior to human milk has yet been discovered, human milk bureaux are a valuable asset in premature-baby care schemes. Extra vitamins are usually started on the seventh day as concentrated preparations, and again are given in three times the normal dosage throughout the period of maximum growth. Most of the small infants develop a fairly severe anaemia and those under 3 lb. usually require blood transfusion, but all should have extra iron from the fourth week.

The care of these babies is largely a matter of strict attention to every small detail, with careful recording and treatment of any abnormality as soon as it arises. Much must rest with the skill of the nurse, who only develops judgment as a result of considerable experience in handling these babies.

The care of the individual baby has been very briefly described, but the service as a whole must now be considered. About five hundred premature babies are born in Bristol every year. How can they best be catered for? Plans for a prematurity scheme started in Bristol in 1942 and 1943. In 1944 a complete report was accepted by the Health Committee for the care of all newborn infants in City Hospitals and especially for premature babies. After seven years, at the beginning of 1951, the whole of that report had been implemented. The hospital prematurity units with their experienced staff, hot rooms and all equipment for emergency treatment will deal with the majority of the babies. Where home conditions are suitable and the babies sufficiently vigorous to be dealt with by relatives between visits from the midwives, special equipment and specially trained midwives are available for the care of larger babies in their own homes. It is essential that a suitably warmed room should be devoted entirely to the mother and baby, and there must be no infection in the household.

Experience has shown that there are three other needs for a complete service for premature babies. Firstly an arrangement whereby premature babies born at home, who are unsuitable for home care or become ill, may be properly transported to hospital and nursed there. The emergency team therefore takes a heated cot, oxygen and resuscitation equipment to the home, bringing the baby back to hospital in a heated ambulance. Secondly the John Milton Mother and Baby Unit has been opened at Southmead Hospital with individual rooms for the isolation of sick premature babies. Here the mothers can be trained in their care and any baby who fails to do well after discharge can be readmitted. A human-milk bureau is the only satisfactory way of ensuring a constant supply of human milk, which is invaluable for feeding most premature babies. A bureau was opened to serve the South Western Region at Southmead Hospital in 1950. Thirdly a follow-up scheme is essential to ensure continued satisfactory care of the babies and to assesss the results of treatment.

The results of treatment of premature babies in Southmead Hospital may be seen in Tables II and III.

From 1946 to 1951, 1,438 premature babies were treated of whom 1,132 were discharged alive; of these 561 were discharged from the special Unit. The total mortality rate was 20 per cent. The majority of babies (366) weighed between 3 lb. 4 oz. and 4 lb. 6 oz. and of these 282 survived—a survival rate of 77·0 per cent. For smaller babies there has been a slight improvement in the survival rate throughout the five-year period and 76 babies were discharged alive weighing less than 3 lb. 4 oz.

TABLE II

*All premature babies treated at Southmead Hospital*

| Year | Premature Unit | | | | Nurseries | | | | All Prematures | | | |
|---|---|---|---|---|---|---|---|---|---|---|---|---|
| | Alive | Dead | Total | Survival rate | Alive | Dead | Total | Survival rate | Alive | Dead | Total | Survival rate |
| | | | | (per cent) | | | | (per cent) | | | | (per cent) |
| 1946 | 67 | 45 | 112 | 60·0 | No statistics available | | | | 67+ | 45+ | 112+ | 60·0 |
| 1947 | 110 | 45 | 155 | 71·0 | 152 | 17 | 169 | 90·0 | 262 | 62 | 324 | 80·9 |
| 1948 | 111 | 42 | 151 | 73·0 | 143 | 12 | 155 | 92·3 | 254 | 54 | 308 | 82·5 |
| 1949 | 129 | 58 | 187 | 69·0 | 131 | 15 | 146 | 89·7 | 260 | 73 | 333 | 78·1 |
| 1950 | 144 | 65 | 208 | 69·4 | 145 | 7 | 152 | 95·4 | 289 | 72 | 361 | 80·0 |
| Total | 561 | 255 | 816 | 68·4 | 571 | 51 | 622 | 92·0 | 1132+ | 306+ | 1438+ | — |

TABLE III

SURVIVAL RATE ACCORDING TO BIRTH WEIGHT. SOUTHMEAD HOSPITAL

*Ministry of Health*, 1951 *Weight Groups*

| Birth Weight | Premature Unit | | | Nurseries | | | All Prematures | | |
|---|---|---|---|---|---|---|---|---|---|
| | Cases | Lived | Survival (%) | Cases | Lived | Survival (%) | Cases | Lived | Survival (%) |
| 2 lb. 3 oz. and under | 41 | 7 | 17·0 | 1 | — | 0 | 42 | 7 | 17·0 |
| 3 lb. 4 oz.—2 lb. 3 oz. | 166 | 64 | 38·5 | 9 | 1 | 11·0 | 175 | 65 | 37·0 |
| 4 lb. 6 oz.—3 lb. 4 oz. | 366 | 282 | 77·0 | 28 | 23 | 82·0 | 394 | 305 | 77·0 |
| 4 lb. 15 oz.–4 lb. 6 oz. | 175 | 151 | 86·0 | 154 | 140 | 91·0 | 329 | 291 | 88·5 |
| 5 lb. 8 oz.—5 lb. | 65 | 56 | 86·0 | 431 | 407 | 94·4 | 496 | 463 | 93·0 |

(9 unweighed babies died in labour wards)

The saving of the lives of premature babies during the first few weeks is costly, particularly in its demand for skilled nursing staff. The scheme might therefore be unprofitable if the infants were subsequently to fall an easy prey to death and disease later in the first year of life, or if, after survival, they were found to be physically or mentally useless individuals. An after-care scheme is therefore essential both for advice on the care of the babies as well as early detection of abnormality and assessment of developmental progress.

Shakespeare described a popular belief about the premature baby in the words of Richard III. " *I, that am rudely stamped, deformed, unfinished, sent before my time into this breathing world scarce half made up and that so lamely and unfashionable that dogs bark at me as I halt by them.*" Fortunately the results of after-care supervision show that this is not so. Most babies, however small at birth, reach the weight of at least 14 lb. by six months provided they are adequately fed. Except for the physiological anaemia of prematurity which occurs in the first few weeks in the very small infant, and is best treated by blood transfusion, the appearance of rickets and anaemia in the first nine months is prevented by good supplements of vitamins and iron. During the second year of life, however, anaemia has developed in a few babies who received inadequate weaning diets owing to poverty or maternal ignorance. Intelligence tests have been carried out on about one quarter of all the babies discharged from the Unit, and these show that the majority of children fall within the average range of intelligence. In 571 babies nursed in the Unit at Southmead, four died before leaving the hospital, but after the first four weeks of age. Two of these children had congenital abnormalities incompatible with continued existence. Of the remaining 567 who left hospital twenty died during the first two years of life, but there have been no deaths over this age. Bronchopneumonia accounted for nine of these deaths, four of them being sudden deaths at home which were reported to the Coroner. Four children who were recognized as aments died from respiratory infections. Infection in some form accounted for all the deaths after discharge from hospital, and except for three deaths after one year, they were evenly distributed throughout the first year of life. The mortality rate therefore in this group of babies after the neonatal period was 3·5 per cent (Table IV), a significantly higher figure than in the rest of the child population in Bristol. Hence the emphasis on the need for special "After-Care" schemes.

TABLE IV

PREMATURE INFANT DEATHS OVER 4 WEEKS

| | |
|---|---|
| In Premature Unit .. .. .. .. .. .. .. .. | 4 |
| After Discharge in 567 survivors (3·5 per cent) .. .. .. .. | 20 |

AGE AT DEATH

| 1–3 *mths.* | 3–6 *mths.* | 6–12 *mths.* | 12–24 *mths.* |
|---|---|---|---|
| 7 | 5 | 9 | 3 |

CAUSE OF DEATH

| | | |
|---|---|---|
| Bronchopneumonia .. .. .. | 9 | (1 before discharge, 3 admitted to hospital, 5 at home, 4 reported to Coroner). |
| Amentia, Bronchitis .. .. .. | 4 | |
| Congenital abnormalities .. .. | 2 | (1 before discharge). |
| Pertussis .. .. .. .. | 2 | |
| Measles .. .. .. .. | 2 | |
| Gastroenteritis .. .. .. | 1 | |
| Chronic Pyelonephritis .. .. | 1 | (before discharge). |
| Convulsions .. .. .. .. | 1 | |
| Marasmus .. .. .. .. | 1 | (reported to Coroner). |
| Congenital Syphilis .. .. | 1 | (before discharge). |

Although it must be emphasized that the majority of children were healthy and normal, a wide variety of abnormalities was encountered in the whole series. The most common defect was ophthalmic, and there were eighteen cases of retrolental fibroplasia. This is probably the most serious problem in premature babies at the present time as it frequently causes total blindness. However, treatment with ACTH at the right stage offers some hope even for this serious condition. The only other problem is cerebral palsy. Eight babies suffered from this condition, most of them very small but sufficiently intelligent to be educable by special methods. There were five babies with congenital hypertrophic pyloric stenosis, all of whom did well, and one with congenital oesophageal atresia who was successfully operated on by Mr. Belsey.

The question is often asked whether very small babies are really worth saving. One can answer this with a most emphatic affirmative in the majority of cases. In this series there were 76 babies weighing less than 3 lb. 4 oz. who left hospital, and 22 of them weighed less than 2 lbs. 12 oz. Of these babies 53 are absolutely normal physically and mentally; six have died; seven have retrolental fibroplasia; five have cerebral palsy; two are mentally deficient; one is hydrocephalic but of normal intelligence; one has congenital cataracts, one has a webbed arm, which causes little disability. The birth weight of the smallest surviving child was 1 lb. 12 oz. (Table 5). Studies on every aspect of the development of premature

TABLE V

RESULTS FOR VERY SMALL BABIES

1946–51. Total Survivors below 3 lb. 4 oz.: 76

| | |
|---|---|
| *Below 2 lb. 12 oz.* | |
| Normal and healthy | 12 |
| Died since discharge | 3 |
| Retrolental fibroplasia | 3 |
| Cerebral Palsy | 3 |
| Webbed arm | 1 |
| TOTAL | 22 |
| *2 lb. 12 oz.—3 lb. 4 oz.* | |
| Normal and healthy | 41 |
| Died since discharge | 3 |
| Retrolental fibroplasia | 4 |
| Cerebral Palsy | 2 |
| Hydrocephalus | 1 |
| Amentia and Blind | 1 |
| Congenital Cataract | 1 |
| Mentally subnormal | 1 |
| TOTAL | 54 |

babies are being continued but the results so far indicate overwhelmingly that all efforts to maintain life for the premature baby are well justified.

This work for premature babies has been achieved by team-work in which many people have co-operated, particularly Professor R. H. Parry and his colleagues of the Public Health Department, of whom special mention must be made of Dr. Greta Hartley, who has taken a considerable part in the clinical work. Essential and much-valued help has been given by all the obstetricians in Bristol, and the nurses, midwives and health visitors associated with various parts of the scheme. Professor Neale has given great help and encouragement since he has been in Bristol. Special tribute must be paid to the nursing staffs of the Unit and above all to the sisters-in-charge, for without their skill and devotion to duty not one of the babies would have survived.

### REFERENCES

De Lee and Greenhill (1947), Philadelphia, *Principles and Practice of Obstetrics*, 767.
Harris, Theodore, (1742) London. *Treatment of Acute Diseases of Infants*, 38.
Price, P. B. (1951). *J. Amer. Med. Assoc.*, **145**, 781.

